# Soft tissue thickness evaluation in screw-retained crowns by the biologically oriented preparation technique (BOPT)

**DOI:** 10.4317/jced.58952

**Published:** 2021-12-01

**Authors:** Victoria Mandillo-Alonso, Rocío Cascos-Sánchez, José-Luis Antonaya-Martín, Martín Laguna-Martos

**Affiliations:** 1 DDS, MDent. Collaborating Professor of Master Prótesis sobre Implantes. Rey Juan Carlos University. Avenida de Atenas s/n, 28922, Alcorcón, Madrid, España; 2 DDS, MSD, MDS. MDent. Collaborating Professor of Postgrade Implantoprótesis Avanzada. Complutense University. Plaza Ramón y Cajal s/n, 28040, Madrid, España; 3 DDS, MSD, MDS. MDent. Collaborating Professor of Master Prótesis sobre Implantes. Rey Juan Carlos University. Avenida de Atenas s/n, 28922, Alcorcón, Madrid, España; 4 DDS, MSD, PhD, MDent. Director of Master Prótesis sobre Implantes. Rey Juan Carlos University. Avenida de Atenas s/n, 28922, Alcorcón, Madrid, España; 5 DDS, MSD, PhD, MDent. Collaborating Professor of Postgrade Implantoprótesis. Complutense University. Plaza Ramón y Cajal s/n, 28040, Madrid, España; 6 DDS. Collaborating Professor of Master Prótesis sobre Implantes. Rey Juan Carlos University. Avenida de Atenas s/n, 28922, Alcorcón, Madrid, España

## Abstract

**Background:**

Intraoral scanner evaluation (3Shape, TRIOS®) of soft tissue thickness around convergent collar implants and shoulderless abutments.

**Material and Methods:**

Ambispective longitudinal analytical study with a sample size of 26 implants in 17 patients treated in a private dental clinic. Pacients were divided into two groups: Prama Implants or group 1 (n=19) and Shelta implants combined with XA abutment or group 2 (n=7). Thickness changes after one- and two-year follow-up were analyzed.

**Results:**

In group 1 baseline mean thickness was 6.53 mm (±1.06) and follow-up mean thickness was 8.06 mm (±0.98), in group 2 initial mean thickness was 7.66 mm (±1.09) and follow-up mean thickness was of 8.42 mm (±1.03).

**Conclusions:**

Biologically guided crowns design seems to significantly increase the soft tissue volumen around convergent morphology implants.

** Key words:**Biologic width, peri-implant soft tissue, marginal bone loss, transmucosal implant, convergent collar, BOPT (biological oriented preparation technique), BOPT abutment, soft tissue stability, intraoral scanner.

## Introduction

Implant treatment success is determined by the integration and stabilization of hard and soft tissues (1,2). Peri-implant soft tissues stability gives a natural appearance to rehabilitation while protecting it from external agents and avoiding bone resorption (3). Recently, new implant abutments and crown designs inspired by the Biological Oriented Preparation Technique (BOPT) have been developed in order to improve insertion of peri-implant soft tissues to avoid bacterial contamination of the alveolar bone (3,4).

This technique is based on a vertically prepared prosthodontic protocol with no finish line which allows the mucosa adaptation to the prosthetic profile determined by the crown (4). Thus, by modifying the contours of the crown, the clinician can control and modify the marginal level of peri-implant soft tissues. On natural teeth, preparation eliminates the anatomical cement-enamel juntion (CEJ) and places the termination line on the crown, not on the tooth. This allows it to create an ideal gingival architecture modulating the emerging profile of the crowns. Same principle could be applied in intramucosal implants restorations with convergent neck and abutment designs, whose objective is to maximize the available space for the soft tissues. A convergent profile allows tissue to migrate coronally to the area of smaller diameter in early stages of healing, creating a thick, stable and more coronal connective seal, below the profile created with restoration. This sets up a protective barrier for soft tissue and peri-implant structures (3-10).

Two areas are defined in this technique: Booster area (BO) or tissue enhacer zone and Prop Tissue up area (PT) or supporting zone of the gingival margin. BO is defined by the convergence of the cervical area of the tooth, implant or abutment and enhaces the thickening and coronal tissue migration. PT belongs to the crown and its funtions are to maintain the gingival margin to prevent collapse and to stop coronal migration of the gingival margin. The slight over-contouring that characterizes BOPT technique delimits a negative pressure area formed by the crown, the lip and the gingival margin. This, together with mechanotransduccion phenomenon helps horizontal thickening of soft tissues over the course of the patient’s life (11).

Volumetric changes in peri-implant soft tissue areas can be evaluated with calipers on the study models, with endodontic needles, with periodontal probes and also with Cone Beam Computed Tomography (CBCT). However, there are other non-invasive techniques such as intraoral scanners, which generate a three-dimensional digital model that can be exported to a Standard Tesselation Language (STL) file. Intraoral scanners allow the clinical to compare the changes in volumen in different clinical situations by overlapping images generated at different times. This is a non-invasive and highly reproductible way to evaluate soft tissue areas (12,13). For all these reasons, the main objective of this research was to evaluate peri-implant soft tissue changes around Prama implants (Sweden Martina®) and XA abutments (Sweden Martina®) observed with a 3Shape TRIOS® scanner. The null hypothesis was that BOPT technique does not increase soft tissue thickness around convergent implants.

## Material and Methods

- Study design and patient selection.

A preliminary ambispective longitudinal analytical study was carried out from June 2017 until September 2020 at a private dental clinic (Instituto Manchego de Implantología y Estética, Alcázar de San Juan, Spain). The study sample consisted of 26 patients (16 women and 10 men) susceptible to implant treatment took part in the research. The average follow-up time was 16 months.

This study has been approved by the Research Ethics Committee (CEI) of the University Rey Juan Carlos de Madrid, with registration number 1510202018220, following the recommendations of the Declaration of Helsinki. All patients were informed of the purpose and characteristics of the study and signed an informed consent after reading it and resolving any pertinent doubts.

Participants were included in the study according to the following inclusion criteria: patients susceptible to implant treatment, patients over 18 years of age, patients treated with Prama (Sweden Martina®) or Shelta implants (Sweden Martina®), patients with a minimum of one year follow-up, patients who have good oral hygiene and motivated to maintain it, single and partial rehabilitations, anterior and posterior area rehabilitations. On the other hand, the exclusion criteria were the following: patients with medical and dental history that make it difficult to place implants, patients with diseases that may affect bone metabolism such as arthritis or osteoporosis, patients with systemic diseases not controlled or polymedicated, smokers of more than ten cigarettes a day and patients with metal allergies.

A clinical and a radiographical study were carried out. Patients were divided into two groups according to the type of implant that has been placed: Prama implants or group 1 (n=19) and Shelta implants or group 2 (n=7). Shelta implants were combined with a convergent intermediate XA abutment (Sweden Martina®).

The following variables were collected: sex, age, implant position, diameter of the implant, implant length, implant type, presence of connective tissue graft, presence of xenograft, antagonist, immediate implant, intermediate abutment, abutment intermediate size and follow-up time.

- Surgical procedure and post-operative care.

All patients were treated by the same operator, M.L.M. Shelta implants placed in the anterior esthetic sector were accompanied by a connective tissue graft (CTG) and bone regeneration therapy with xenograft (Bio-Oss®, Geistlich Pharma AG) in the gap. Prama implants were placed in the posterior sector and no renegerative theraphy was performed. All Prama implants were placed in a bone level position in order to let the convergent neck to the soft tissue. All Shelta implants were placed in a subcrestal position (1-2 mm). All the implants were placed with a minimum insertion torque of 30 N.

In type I and II sockets, tooth extraction and implant placement were carried out in the same surgical act, that is, “immediate implant placement”.

Patients were medicated with Amoxicillin/clavulanic 875/125 mg one dose every 8 hours for a week and with ibuprofen 600 mg one dose every 8 hours in case of pain. In addition, after the first 24 hours, they were prescribed a 0.2% chlorhexidine rinse for a week at night.

After surgery, all patients attended a review at one week, one month and, finally, at three or five months in order to take impressions for the definitive crown. Average osseointegration time was 5 months.

- Restorative treatment.

Prama implants were rehabilitated with a customized, screwed healing cap the same day of surgery (A-MPSCI-330-EX, Sweden Martina®). The customized immediate healing cap was made with flowable composite following socket anatomy. Shelta implants were rehabilitated with a screw-retained immediate aesthetic provisional (SH-CTABU-F-380, Sweden Martina®) made from a previous wax-up. The objetive of provisionalizing the same day of the surgery was to preserve the clot stability at the sime time that the soft tissue healed according to the shape of the provisional.

**Figure 1:**
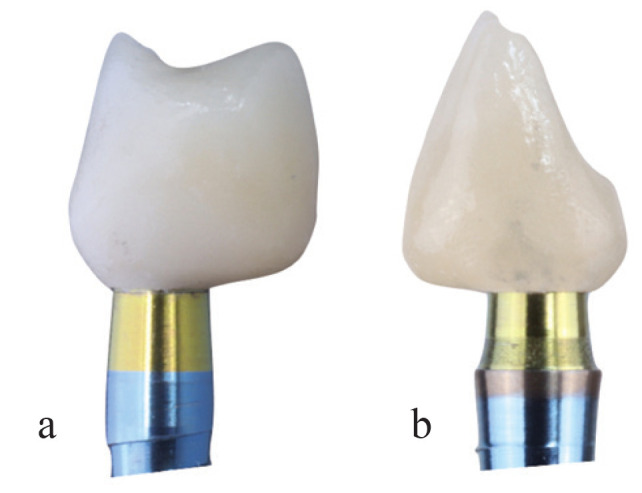
a. Prama restoration. b. Shelta-XA abutment restoration.

Finaly, after osseointegration time and soft tissue modeling, digital impresions were taken using a 3Shape TRIOS® intraoral scanner to make definitive prosthesis. All crowns were screw-retained implant supported made with milled Cr-Co metal and feldespathic ceramic following a BOPT design, which is 1 or 1.5 mm below the gingival margin in order to simulate the natural emergence profile of the teeth. Crowns were made by CAD/CAM design software. In the posterior area, all crowns embrace 0.8 mm of the Prama implant convergent neck to increase stability (Fig. 1a). Shelta implant and XA abutment are represented in Figure 1b. Screw access channel were covered by teflon and composite.

- Data collection.

Data was collected by a single operator, V.M.A, except the initial intraoral scanning procedures which were carried out by M.L.M, second operator.

Data collected was: clinical history; initial frontal, lateral and occlusal intraoral photographs; initial STL files and follow-up STL files.

- Soft tissue thickness evaluation.

**Figure 2:**
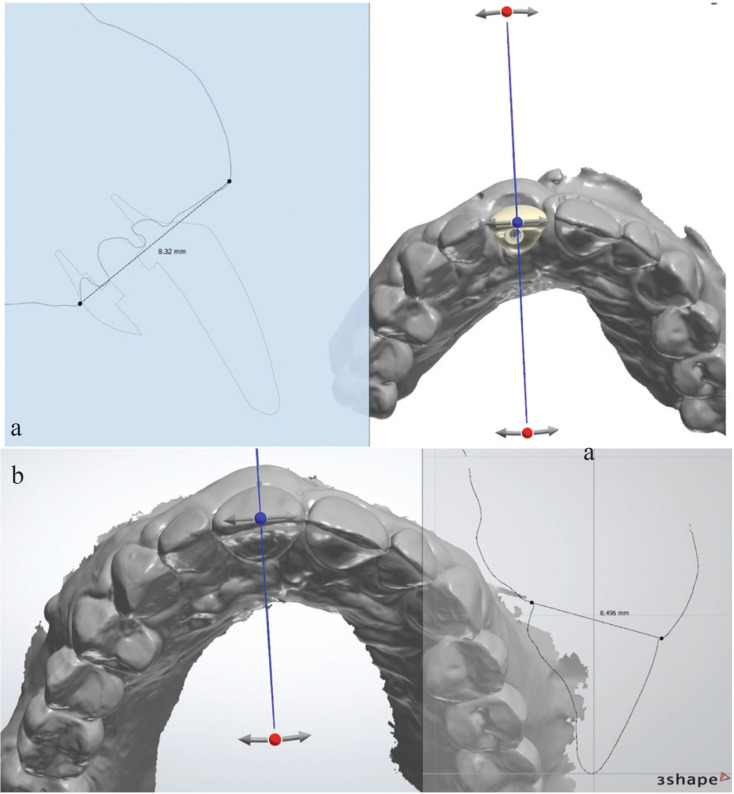
a. Implant initial intraoral scanner measurements (8.32 mm). b. Implant follow-up intraoral scanner measurements (8.496 mm).

In order to evaluate soft tissue thickness, initial measurements, taken the day of definitive crown prints (Fig. 2a), were compared with those taken at the one- or two-year follow-up appointment (Fig. 2b). STL files were processed and analyzed using the 3Shape TRIOS® intraoral scanner software. Two sagittal reference points were chosen for the measurements: the most coronal point of the buccal groove and the most coronal point of the palatal or lingual groove. A horizontal line was drawn to connect both points and then the exact measurement of the soft tissue in millimeters (mm) was taken.

Scanner calibration was carried out before each scan. Scan were taken with the patients sitting in the dental chair with the backrest slightly reclined forming an angle of approximately 110º respect to the ground and with the equipment lights off, only with dental room lights, which means 1000 lux. Scanning started in occlusal and palatal faces followed by vestibular ones. It ended with two bite records, right and left. No additional light source was used, only the light from the scanning system itself.

- Statistical analysis of the data.

Statistical analysis was carried out using the SPSS Statistics version 25 software application (IBM; Armonk. NY, USA), using the Student’s t-test for repeated measures in the contrast of variables between the initial and control averages and between groups of cases independent from each other.

## Results

The study sample consisted of 26 implants. Table 1 summarizes the characteristics of the implants. Implants were classified in two groups: group 1 (n=19) included Prama implants, without connective tissue graft and without xenograft, and group 2 (n=7) included Shelta implants, with connective tissue graft and with xenograft. All Shelta implants were combined with XA abutments: 4 mm-abutment in 42.9%, 5 mm-abutment in 28.6% and 6 mm-abutment in 28.5%.

**Table 1:**
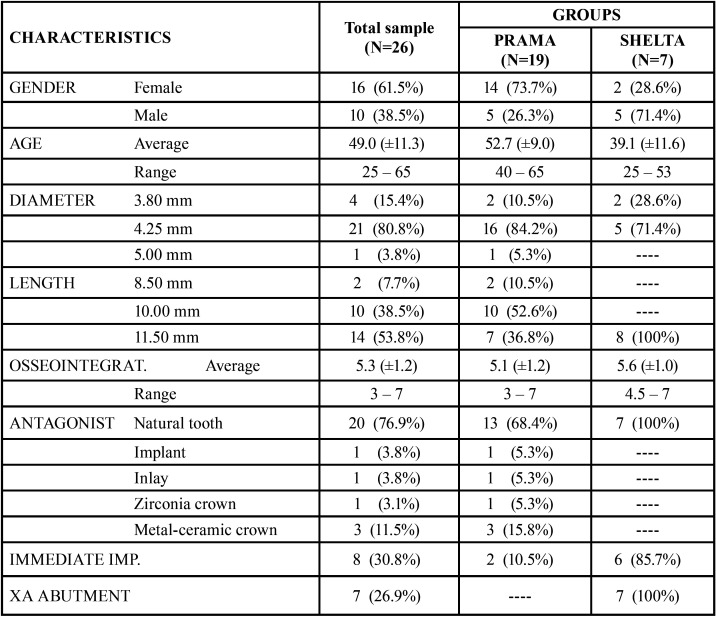
Descriptive analysis. Sample characteristics.

A total of 16 implants (61.5%) were placed in women and 10 implants (38.5%) were placed in men. The age of the patients ranged between 25 and 65 years old with an average age of 49 years (±11.3). In this sample, 7 implants (29.6%) were placed in the anterior area (incisors or canines) and 19 implants (73.1%) were placed in the posterior area (molars or premolars). Osseointegration time ranged between 3 and 7 months, with an average time of 5.3 months (± 1.2). Most of implant antagonists were natural pieces (76.9%). 23.1% of antagonists were implants, inlays, zirconia crowns or metal-ceramic crowns. 30.8% of the implants were placed immediately after extraction.

- Soft tissue thickness.

**Figure 3:**
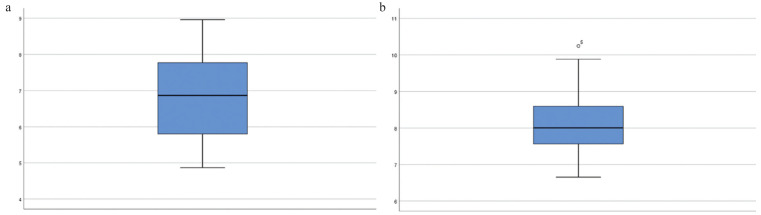
a. Soft tissue initial measurements (range of 4.87 - 8.96 mm). b. Soft tissue follow-up measurements (range of 6.66 - 10.24 mm).

Values obtained from both groups in initial measurements (Fig. 3a) oscillated in the range of 4.87-8.96 mm and the initial average was 6.84 mm (±1.16). Values obtained from both groups in follow-up measurements (Fig. 3b) ranged between 6.66-10.24 mm and the follow-up average was 8.16 mm (± 0.99).

The difference between the initial average (6.84 mm) and the follow-up average (8.16 mm) was contrasted using the Student’s t-test for repeated measures, resulting in a highly significant change (*p*<0.001) accompanied by a significant effect size (0.793 mm). Variation of soft tissue thickness is reflected in Table 2. We found that changes remained very significant and with very high effect sizes (around 0.84 mm) in both groups.

**Table 2:**
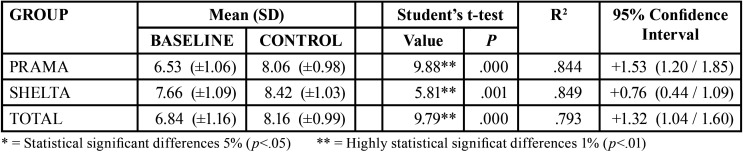
Variation of the soft tissue thickness.

Differences between initial and follow-up measurements are greater in Prama implants (1.53 mm) than in Shelta implants (0.76 mm) with high statistical significance (*p* <0.01).

-Effect of factors on the soft tissue thickness.

• Gender.

Soft tissue thickness in men (0.95 mm) is statistically significantly greater (*p*<0.01) than in women (0.31).

• Age.

Magnitude of soft tissue thickness is higher among cases of age over 52 years (10.5 mm vs 0.65 mm). Although this difference does not reach statistical significance (*p*> 0.05), effect size (moderate: 0.306) indicates a possible relationship that would imply an effect of age on soft tissue thickness.

• Antagonist.

Identical thickness was found regardless of the antagonist. Therefore, it could be concluded that this variable is not a factor related to soft tissue thickness.

• Immediate implant placement.

Average soft tissue thickness observed in those cases in which an immediate implant was placed (0.86 mm) was higher than in those that were not performed (0.18 mm). Although this difference does not reach statistical significance (*p*>0.05), effect size (0.557) it is reasonably enough to consider that there could be a remarkable relationship that suggests that the placement of an immediate implant influences soft tissue thickness.

## Discussion

New implant designs, abutments and crowns inspired by the BOPT on teeth improve insertion of the peri-implant soft tissues in order to avoid bacterial contamination. We know that connective tissue forms a protective barrier around implants or intermediate abutments. When connective tissue stabilizes, it prevents apical migration of the junctional epithelium and determines the amount of bone resorption. (14). The objective of this research was to evaluate peri-implant soft tissue changes around Prama impants and XA abutments. The null hypothesis was that BOPT technique does not increase soft tissue thickness around convergent implants.

It is worth noting the role of the keratinized gingiva thickness and the peri-implant mucosa. Keratinized gingiva is defined as the height of the soft tissue that runs in an apico-coronal direction from the gingival margin to the mucogingival line (15). An insufficient amount of keratinized gingiva (less than 2 mm) is associated with plaque, inflammation, recessions, and attachment loss. That means mucositis which, maintained over time, can lead to peri-implantitis (16,17). Peri-implant mucosa is defined as the horizontal dimension of the peri-implant soft tissue (15) and may play an important role in the functional and aesthetic results of implant therapy, as well as in the maintenance of peri-implant health. Thin soft tissues can cause loss of crestal bone during the formation of the peri-implant sealing, which entablished that we need a minimum of 2 mm of peri-implant mucosa (15-17).

Peri-implant mucosa consists of the junctional epithelium and the connective tissue, both ensure an optimal sealing around implants and provide protection against biological and mechanical external agents (18). When an external agent damages the biological space, the epithelium responds by migrating beyond the damaging agent in an attempt to isolate so, in the end, connective tissue is exposed, with the consequent demineralization and bone resorption (14).

Peri-implant soft tissue volumentric changes can be evaluated with non-invasive techniques such as intraoral scanners that generate 3D images in different moments in a highly reproducible way (12,13). Our goal is to evaluate the soft tissue thickness around Prama implants and Shelta implants with XA abutments observed with a 3Shape TRIOS® intraoral scanner.

In the present study, a significant increase of the soft tissue volume has been observed in Prama and Shelta implants made with a biologically guided crown. Regarding Prama implants, the initial average values were 6.53 (±1.06) and the follow-up values were 8.06 (±0.98). The initial average values of the Shelta group were 7.66 (±1.09) and the follow-up values were 8.42 (±1.03). Therefore, Prama group presented a greater increase in soft tissue volume than Shelta group. Acording to these results the hypothesis null that BOPT technique does not increase soft tissue thickness around convergent implants was rejected.

Sanz *et al*. (2) observed volumetric changes of soft tissue around immediate implants and delayed implants in a study in dogs with a follow-up period of 12 weeks. They concluded that soft tissue volume in immediate implants group was slightly higher. In our study we have observed a highly significant volumetric changes in immediate implant cases. These results may be due to the fact that when we perform an immediate implant placement, the gap is filled with a sTable clot that will turn into a thick and sTable soft tissue.

Regarding to the convergent morphology of the Prama implant and the XA abutment, Rompen *et al*. (19) declared that the use of concave transmucosal profiles seems to allow predicTable and better soft tissue stability in aesthetic areas than divergent profiles. Canullo *et al*. (7) advocate that the use of a BOPT protocol with convergent neck tissue level implants maintains the stability of the soft tissue after 3 years of follow-up. Agustín *et al*. (8) declare that implants with convergent neck designs have less marginal bone loss compared to implants of divergent neck designs. Cabanes *et al*. (3) agree that the placement of crowns with a BOPT design with convergent neck abutments results in sTable soft tissue. They even point out in his study that after 10 months from the loading of the prosthetic restorations there was an increase in the soft tissue volume. However, their clinical protocol differs from our study because a provisional was not placed the same day of surgery to stabilize the clot. It would be interesting to evaluate the same parameters with the same characteristics, but performing an immediate provisional.

Although our study describes only soft tissue thickness around convergent abutments and implants there are many authors who report a good thickness of peri-implant soft tissue with the prevention of marginal bone loss (3,6,8-11,13,14). Agustín *et al*. (9) made a study to evaluate the behavior of the soft tissue around conventional screw-retained crowns, conventional cemented crowns and BOPT cemented crowns. They conclude saying that cemented BOPT crowns obtain better keratinized gingiva, less probing depth and lower incidence of bleeding on probing than screw-retained crowns or conventional cemented crowns. Therefore, there is a direct correlation between soft tissue and marginal bone loss: the better keratinized gingiva we have, the less marginal bone loss, the less probing depth and the less bone loss.

The present study had some limitations. Despite these significant results that directly report an increase of the soft tissue area with the biologigally oriented preparation technique, it is necessary to clarify that the sample size is small and the follow-up time is short. However, we have obtained promising results.

In conclusion, design of biologically guided crowns seems to significantly increase soft tissue thickness around implants of convergent morphology. However, further studies are needed to support the findings of the present study. It is necessary to extend the sample size and the follow-up time in order to obtain more significant results.

## References

[1] Branemark PI (1983). Osseointegration and its experimental back-ground. Journal of Prosthetic Dentistry.

[2] Sanz-Martin I, Vignoletti F, Nuñez J, Permuy M, Muñoz F, Sanz-Esporrín J (2017). Hard and soft tissue integration of immediate and delayed implants with a modified coronal macrodesign: Histological, micro-CT and volumetric soft tissue changes from a pre-clinical in vivo study. J Clin Periodontol.

[3] Cabanes-Gumbau G, Pascual-Moscardó A, Peñarrocha-Oltra D, García-Mira B, Aizcorbe-Vicente J, Peñarrocha-Diago MA (2019). Volumetric variation of peri-implant soft tissue in convergent collar implants and crowns using the biologically oriented preparation technique (BOPT). Med Oral Patol Oral Cir Bucal.

[4] Loi I, Scutella F, Galli F (2008). Technique of biologically oriented preparation (BOPT). A new approach to prosthetic preparation in odontostomatology. Quintessenza Internazionale.

[5] Loi I, Di Felice A (2013). Biologically oriented preparation technique (BOPT): a new approach for prosthetic restoration of periodontically healthy teeth. Eur J Esthet Dent.

[6] Serra-Pastor B, Loi I, Fons-Font A, Solá-Ruíz MF, Agustín-Panadero R (2019). Periodontal and prosthetic outcomes on teeth prepared with biologically oriented preparation technique: a 4-year follow-up prospective clinical study. J Prosthodont Res.

[7] Canullo L, Menini M, Covani U, Pesce P (2020). Clinical outcomes of using a prosthetic protocol to rehabilitate tissue-level implants with a convergent collar in the esthetic zone: A 3-year prospective study. J Prosthet Dent.

[8] Agustín-Panadero R, Bustamante-Hernández N, Solá-Ruíz MF, Zubizarreta-Macho Á, Fons-Font A, Fernández-Estevan L (2019). Influence of Biologically Oriented Preparation Technique on Peri-Implant Tissues; Prospective Randomized Clinical Trial with Three-Year Follow-Up. Part I: Hard Tissues. J Clin Med.

[9] Agustín-Panadero R, Bustamante-Hernández N, Labaig-Rueda C, Fons-Font A, Fernández-Estevan L, Solá-Ruíz MF (2019). Influence of Biologically Oriented Preparation Technique on Peri-Implant Tissues; Prospective Randomized Clinical Trial with Three-Year Follow-Up. Part II: Soft Tissues. J Clin Med.

[10] Díaz-Sánchez M, Soto-Peñaloza D, Peñarrocha-Oltra D, Peñarrocha-Diago M (2019). Influence of supracrestal tissue attachment thickness on radiographic bone level around dental implants: A systematic review and meta-analysis. J Periodontal Res.

[11] Rodríguez X, Vela X, Segalà M, Pérez J, Pons L, Loi I (2019). Human histological examination of tissue response to vertical grinding and immediate provisionalization (Biological Basis BOPT). Clinical periodontics and restorative dentistry.

[12] Tavelli L, Barootchi S, Majzoub J, Siqueira R, Mendonça G, Wang HL (2021). Volumetric changes at implant sites: A systematic appraisal of traditional methods and optical scanning-based digital technologies. J Clin Periodontol.

[13] Sanz Martin I, Benic GI, Hämmerle CH, Thoma DS (2016). Prospective randomized controlled clinical study comparing two dental implant types: volumetric soft tissue changes at 1 year of loading. Clin Oral Implants Res.

[14] Matta Valdivieso, Alarcon Palacios, Matta Morales (2012). Espacio biológico y prótesis fija: Del concepto clásico a la aplicación tecnológica. Rev Estomatol Herediana.

[15] Avila-Ortiz G, Gonzalez- Martin O, Couso-Queiruga E, Wang HL (2020). The peri-implant phenotype. J Periodontol.

[16] Tavelli L, Barootchi S, Avila-Ortiz G, Urban IA, Giannobile WV, Wang HL (2021). Peri-implant soft tissue phenotype modification and its impact on peri-implant health: A systematic review and network meta-analysis. J Periodontol.

[17] Longoni S, Tinto M, Pacifico C, Sartori M, Andreano A (2019). Effect of Peri-implant Keratinized Tissue Width on Tissue Health and Stability: Systematic Review and Meta-analysis. Int J Oral Maxillofac Implants.

[18] Linkevicius T, Apse P (2008). Biologic width around implants. An evidence-based review. Stomatologija.

[19] Rompen E, Raepsaet N, Domken O, Touati B, Van Dooren E (2007). Soft tissue stability at the facial aspect of gingivally converging abutments in the esthetic zone: a pilot clinical study. J Prosthet Dent.

